# Supplementary material for: Reliability assessment of temporal discounting measures in virtual reality environments
Source: Sci Rep. 2021 Mar 29;11:7015. doi: 10.1038/s41598-021-86388-8 (PMC8007609; doi:10.1038/s41598-021-86388-8)
Supplement: Supplementary file 1 — Supplementary Information 1. [file 41598_2021_86388_MOESM1_ESM.pdf]

Reliability assessment of temporal discounting measures in virtual reality environments

Luca R. Bruder*, Lisa Scharer, Jan Peters

Department of Psychology, Biological Psychology, University of Cologne, Germany

*** Corresponding author**

Contact: lbruder@uni-koeln.de, jan.peters@uni-koeln.de

Supplementary Materials

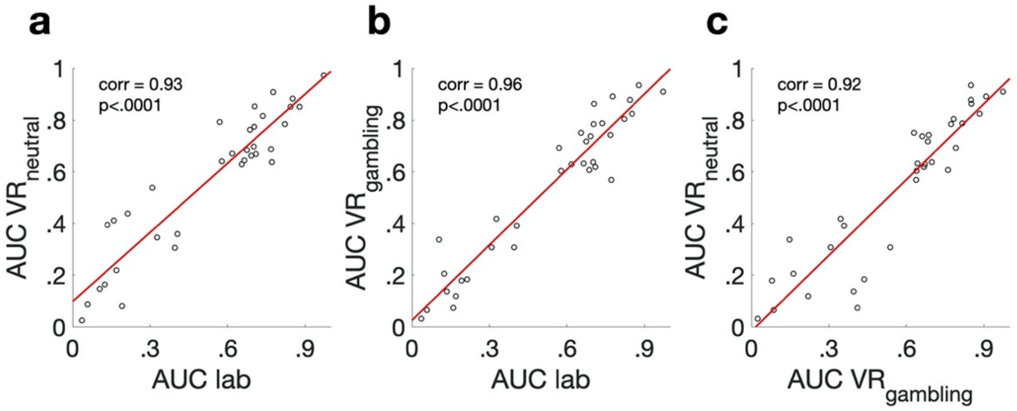

Supplementary Figure S1. Scatterplots of the individual participants AUC values. a) lab vs $VR_{neutral}$ b) lab vs $VR_{gambling}$ c) $VR_{gambling}$ vs $VR_{neutral}$.

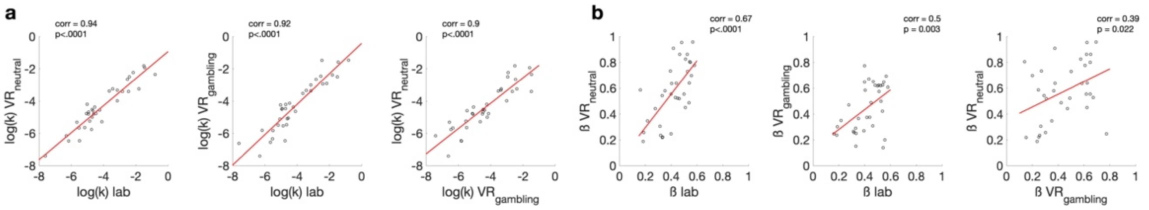

Supplementary Figure S2. Scatterplots of the mean of the individual participants parameter posterior distributions for the parameters of the hyperbolic discounting model with the softmax choice rule (see equation 1 and 2). a) *log(k)* b) softmax ß.

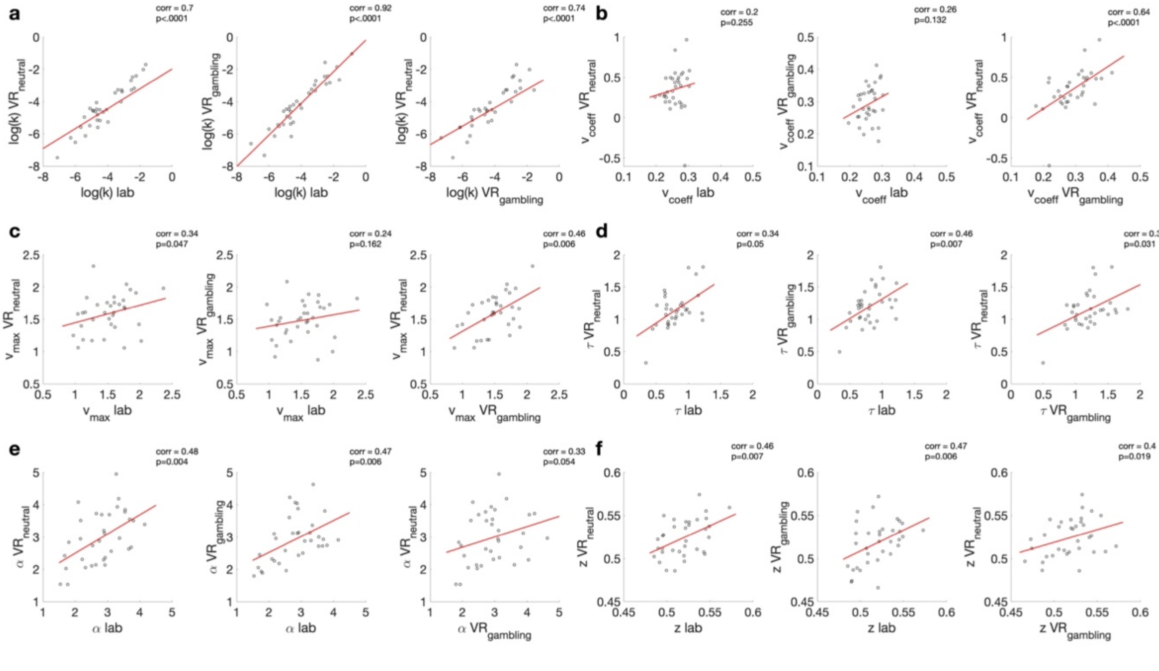

Supplementary Figure S3. Scatterplots of the mean of the individual participants parameter posterior distributions for the parameters of the DDM_S temporal discounting model. a) *log(k)* b) v_{coeff} c) v_{max} d) tau e) α f) z.

Supplementary Table S1. Means and 95% HDIs of the posteriors of the parameters from the DDM_s model.

Session	Log(k)			vcoeff			vmax			τ			α			z		
	Mean	HDI		Mean	HDI		Mean	HDI		Mean	HDI		Mean	HDI		Mean	HDI	
**Lab**	-4.051	-4.57	-3.54	.26	.22	.306	1.516	1.362	1.677	.806	.724	.889	2.806	2.545	3.073	.519	.502	.537
**$VR_{neutral}$**	-4.474	-5.012	-3.95	.352	.238	.491	1.588	1.428	1.756	1.145	1.041	1.249	2.978	2.671	3.293	0.524	.506	.542
**$VR_{gambling}$**	-4.16	-4.698	-3.626	.292	.244	.351	1.484	1.342	1.636	1.197	1.099	1.294	2.921	2.648	3.199	.518	.499	.537
**Lab-$VR_{neutral}$**	.422	-.309	1.16	-.093	-.237	.0305	-.072	-.3	.157	-.339	-.471	-.207	-.173	-.585	0.236	-.005	-.03	.02
**Lab-$VR_{gambling}$**	.108	-.628	.849	-.033	-.104	.034	.032	-.181	.247	-.391	-.516	-.265	-.115	-.497	0.266	.001	-.025	.027
**$VR_{gambling}$-$VR_{neutral}$**	.314	-.434	1.072	-.06	-.208	.069	-.104	-.324	.115	.052	-.091	.194	-0.058	-.473	.357	-.006	-.032	.02

Supplementary Table S2. Summary of the results of the ICC analysis of the DDM_L parameters.

Parameter	ICC	p	Lower bound	Upper Bound
log(k)	.92	<.001	.88	.95
v_{coeff}	.65	<.001	.5	.77
τ	.15	.067	-.014	.35
α	.36	<.001	.19	.55
z	.60	<.001	.45	.74

Supplementary Table S3. Summary of the results of the split-half ICC analysis of the DDM_S parameters within the lab-session.

Parameter	ICC	p	Lower bound	Upper Bound
log(k)	.97	<.001	.96	.97
v_{coeff}	.25	.069	-.029	.5
v_{max}	.76	<.001	.61	.86
τ	.92	<.001	.86	.95
α	.94	<.001	.9	.97
z	.48	.002	.23	.67

Supplementary Table S4. Summary of the results of the split-half ICC analysis of the DDM_S parameters within the $VR_{neutral}$-session.

Parameter	ICC	p	Lower bound	Upper Bound
log(k)	.73	<.001	.57	.84
v_{coeff}	.005	.49	-.092	.29
v_{max}	.65	<.001	.46	.79
τ	.94	<.001	.9	.97
α	.9	<.001	.82	.94
z	.25	.075	-.036	.49

Supplementary Table S5. Summary of the results of the split-half ICC analysis of the DDM_S parameters for the parameters within the $VR_{gambling}$-session.

Parameter	ICC	p	Lower bound	Upper Bound
**log(k)**	.96	<.001	.56	.8
$\boldsymbol{v}_{\mathbf{coeff}}$	-.1	.73	-.053	.3
$\boldsymbol{v}_{\mathbf{max}}$	.58	<.001	.16	.52
$\boldsymbol{\tau}$	.94	<.001	.019	.38
$\boldsymbol{\alpha}$	.92	<.001	.24	.59
**z**	.36	.016	.22	.58
